# The combination of sport and sport-specific diet is associated with characteristics of gut microbiota: an observational study

**DOI:** 10.1186/s12970-019-0290-y

**Published:** 2019-05-03

**Authors:** Lae-Guen Jang, Geunhoon Choi, Sung-Woo Kim, Byung-Yong Kim, Sunghee Lee, Hyon Park

**Affiliations:** 1 Exercise Nutrition & Biochemistry Lab., Kyung Hee University, Yongin, Republic of Korea; 2 Growth and Aging Lab., Kyung Hee University, Yongin, Republic of Korea; 3 ChunLab, Inc., Seocho-gu, Seoul Republic of Korea; 4 Research Lab., Ildong Pharmaceutical Co., Ltd., Hwaseong, Republic of Korea

**Keywords:** Gut microbiota, Body builder, Distance runner, Dietary fiber

## Abstract

**Background:**

Recently, gut microbiota have been studied extensively for health promotion, disease prevention, disease treatment, and exercise performance. It is recommended that athletes avoid dietary fiber and resistant starch to promote gastric emptying and reduce gastrointestinal distress during exercise, but this diet may reduce microbial diversity and compromise the health of the athlete’s gut microbiota.

**Objective:**

This study compared fecal microbiota characteristics using high-throughput sequencing among healthy sedentary men (as controls), bodybuilders, and distance runners, as well as the relationships between microbiota characteristics, body composition, and nutritional status.

**Methods:**

Body composition was measured using DXA, and physical activity level was assessed using IPAQ. Dietary intake was analyzed with the computerized nutritional evaluation program. The DNA of fecal samples was extracted and it was sequenced for the analysis of gut microbial diversity through bioinformatics cloud platform.

**Results:**

We showed that exercise type was associated with athlete diet patterns (bodybuilders: high protein, high fat, low carbohydrate, and low dietary fiber diet; distance runners: low carbohydrate and low dietary fiber diet). However, athlete type did not differ in regard to gut microbiota alpha and beta diversity. Athlete type was significantly associated with the relative abundance of gut microbiota at the genus and species level: *Faecalibacterium*, *Sutterella*, *Clostridium*, *Haemophilus,* and *Eisenbergiella* were the highest (*p* < 0.05) in bodybuilders, while *Bifidobacterium* and *Parasutterella* were the lowest (*p* < 0.05). At the species level, intestinal beneficial bacteria widely used as probiotics (*Bifidobacterium adolescentis group*, *Bifidobacterium longum group, Lactobacillus sakei group*) and those producing short chain fatty acids (*Blautia wexlerae, Eubacterium hallii*) were the lowest in bodybuilders and the highest in controls. In addition, aerobic or resistance exercise training with an unbalanced intake of macronutrients and low intake of dietary fiber led to similar diversity of gut microbiota. Specifically, daily protein intake was negatively correlated with operation taxonomic unit (r = − 0.53, *p* < 0.05), ACE (r = − 0.51, *p* < 0.05), and Shannon index (r = − 0.64, *p* < 0.01) in distance runners..

**Conclusion:**

Results suggest that high-protein diets may have a negative impact on gut microbiota diversity for athletes, while athletes in resistance sports that carry out the high protein low carbohydrates diet demonstrate a decrease in short chain fatty acid-producing commensal bacteria.

## Introduction

Regular exercise offers a beneficial effect on health and a preventive effect on non-communicable diseases by challenging systemic homeostasis [1]. Practically, exercise is recommended as a useful tool to prevent disease and improve the prognosis when an athlete becomes sick or injured. Diseases in which exercise produces a preventive and treatment effect include colon and breast cancer [2, 3], type 2 diabetes [4], sarcopenia [5], cardiovascular diseases [6], and stress-related disorders such as anxiety and depression [7]. Recently, gut microbiota are being studied extensively to understand their effects on health promotion, disease prevention and treatment, and how exercise can modulate these effects [8, 9]. For instance, animal studies have indicated that exercise-induced changes in gut microbiota may be involved in the modulation of high fat-induced obesity [10, 11], polychlorinated biphenyls-induced dysbiosis [12], metabolic syndrome [13], experimental diabetes [14], and chemically-induced colitis [15]. Human studies have also reported that regular exercise plays a beneficial role in host health by affecting the structure and diversity of gut microbiota. For example, subjects with higher cardiorespiratory fitness showed high gut microbiota diversity and a relative abundance of butyrate-producing bacteria, which are important in gut microbiota homeostasis [16, 17]. In particular, high-protein intake with exercise training increased the diversity of gut microbiota in a study comparing the gut microbiota of male rugby players and healthy controls [18].

Clark and Mach (2016) reported that diets recommended for athletes likely influence gut microbiota by reducing diversity because many athletes’ diets have insufficient dietary fiber [19]. It is recommended that athletes consume a high amount of monosaccharides to maximize glycogen storage and sustain blood glucose during exercise training, as well as minimize intake of dietary fiber and resistant starch to prevent gastrointestinal disturbances [20]. Low intake of dietary fiber and resistant starch may lead to decreased bowel movements resulting in decreased bowel function, and also decrease the diversity of gut microbiota [21]. In addition, athletes consume more animal protein than non-athletes to satisfy muscle accretion needs [22]. Excessive protein ingestion leads to an excess of nitrogen substrates in the intestinal microbes, producing putrefactive fermentation products such as ammonia, hydrogen sulfide, amines, phenols, thiols, and indoles [23]. As digesta moves through the intestines, the carbohydrate content decreases, and putrefactive fermentation becomes more harmful [24]. In fact, high protein intake is reported to lead to DNA damage in the colon mucosa when dietary levels of fermentable carbohydrates are low [25–27].

Moreover, high-intensity exercise stimulates redistribution of blood from the intestinal organs to the muscles while they actively undergo cellular respiration [28]. The frequent redistribution of blood could potentially disturb gut microbiota by splanchnic hypo-perfusion and ischemia and subsequent reperfusion [29]. Therefore, to investigate the long-term effects of a specific exercise type and athletes’ diets on gut microbiota, we compared the gut microbiota characteristics, dietary intake, and body composition of healthy men in their twenties who did not have previous exercise habits (control group) with those of athletes in their twenties (bodybuilders and distance runners) who adhered to specific exercise training regimes and diets.

## Materials and methods

### Subject characteristics and sample treatments

Bodybuilders (*n* = 15), elite distance runners (*n* = 15), and healthy men in their twenties without regular exercise habits (*n* = 15) were recruited for this study. All subjects were male. Bodybuilders were 25 (±3) years old on average and had athletic careers of 7.6 (±3.7) years. Their mean body mass index (BMI) was 28.1 (±2.6) kg/m^2^. Distance runners were 20 (±1) years old on average and had been runners for 7.5 (±2.1) years. Their mean BMI was 20.5 (±0.8) kg/m^2^. Healthy men without exercise habits were 26 (±2) years old on average and had a mean BMI of 25.9 (±4.2) kg/m^2^.

All subjects provided written informed consent prior to beginning the study. This study was conducted after approval was obtained from the Institutional Review Board of Kyung Hee University. Exclusion criteria were prescribed antibiotics within 6 months, immune diseases, digestive tract disorders, acute or chronic cardiovascular diseases, and metabolic disorders. Fecal samples were collected from all participants as the first process of the stuidy. DNA was extracted from fresh stool samples stored on ice, and samples were frozen at − 80 °C immediately after that.

### Body composition and physical activity level

Body composition of all subjects was measured using dual-energy X-ray absorptiometry (DXA: Hologic, QDR-4500 W, USA) (Table 1). The DXA was calibrated daily with a phantom, and the coefficient of variance was maintained at less than 1.5%. Each participant wore comfortable clothes without any metal. Whole body scanning was performed for 7 min and the results were analyzed by a technician.

**Table 1:** Subject characteristics

	Control (*n* = 15)	Bodybuilders (*n* = 15)	Distance runners (*n* = 15)
Age (years)	26.3 ± 2.0	24.9 ± 2.7	19.8 ± 0.8
Career (years)		7.6 ± 3.7	7.5 ± 2.1
BMI (kg/m^2^)	25.9 ± 4.2^+^	28.1 ± 2.6***	20.5 ± 0.9^¥¥¥^
Lean tissue (kg)	56.5 ± 4.6^+++^	70.4 ± 9.2***	51.8 ± 4.1^¥¥^
Fat tissue (kg)	19.4 ± 7.9^++^	11.9 ± 4.5***	5.5 ± 1.1^¥¥¥^
Body fat %	23.9 ± 6.9^+++^	14 ± 5.1***	9.2 ± 1.6^¥¥¥^

Data shown as mean ± SD, controls versus bodybuilders: ^+^*p* < 0.05 or ^++^*p* < 0.01 or ^+++^*p* < 0.001, bodybuilders versus distance runners: ***p < 0.001, distance runners versus controls: ^¥¥^*p* < 0.01 or ^¥¥¥^*p* < 0.001

*BMI* body mass index

Physical activity level was assessed using the International Physical Activity Questionnaire (IPAQ). The Korean version of the IPAQ questionnaire (http://www.ipaq.ki.se) was used and physical activity level was calculated by Metabolic Equivalent of Task (MET) as described in a previous study [30]. In our study, the average physical activity of the subjects in control sedentary group was 860 (±979) METs during their previous 6 months.

### Dietary intake data collection

Dietary intake information was obtained from each individual based on a 3-day food diary (2 weekdays and 1 weekend day) that reflected habitual dietary intake. Although self-recorded estimates of food intake in food diaries may not provide accurate or unbiased estimates of a person’s energy intake, the participants in our study were supervised by a specialist to ensure that accurate information was provided. Furthermore, macronutrient and micronutrient supplements were recorded. Daily nutrient intake was analyzed using the nutritional evaluation program CANPro 4.0 and Dietary Reference Intakes for Koreans developed by the Korean Nutrition Society [31].

### DNA extraction and high-throughput amplicon sequencing

The DNA of fecal samples was extracted from feces using a Fast DNA™ SPIN extraction kit (MP Biomedicals, Solon, Ohio, USA). The first PCR amplification was performed using a T100 thermal cycler (Bio-Rad, Hercules, CA, USA) to amplify the V3 and V4 regions of 16S rRNA. Primers used were 341F (5′-TCGTCGGCAGCGTC-AGATGTGTATAAGAGACAG-CCTACGGGNGGCWGCAG-3′, the underlined sequence indicates the target region primer) and 805R (5′-GTCTCGTGGGCTCGG-AGATGTGTATAAGAGACAG-GACTACHVGGGTATCTAATCC-3′). The first PCR amplification was carried out under the following conditions: initial denaturation at 95 °C for 3 min followed by 25 cycles of denaturation at 95 °C for 30 s, primer annealing at 55 °C for 30 s, and extension at 72 °C for 30 s, with a final elongation at 72 °C for 5 min. The second PCR amplification to attach the Illumina NexTera barcodes was performed with the i5 forward primer (5′-AATGATACGGCGACCACCGAGATCTACAC-XXXXXXXX-TCGTCGGCAGCGTC-3′, X indicates the barcode region) and the i7 reverse primer (5′-CAAGCAGAAGACGGCATACGAGAT-XXXXXXXX-AGTCTCGTGGGCTCxGG-3′). Conditions used for the second amplification reaction were the same as those described for the first reaction except only eight amplification cycles were performed. Amplification was confirmed by 2% agarose gel electrophoresis and visualization of the PCR products using a Gel Doc system (BioRad). PCR amplification products were purified using a QIAquick PCR purification kit (Qiagen, Valencia, CA, USA). Equal concentrations of purified products were pooled together and short fragments (non-target products) were removed with Ampure beads (Agencourt Bioscience, MA, USA). The size and quality of the amplified product were assessed on a Bioanalyzer 2100 (Agilent, Palo Alto, CA, USA) using a DNA 7500 chip. Mixed amplicons were pooled and sequencing was performed by ChunLab, Inc. (Seoul, Korea) using the Illumina MiSeq Sequencing system (Illumina, USA).

### Bioinformatics analysis

Raw reads were quality checked and low-quality reads (<Q25) were filtered out using trimmomatic 0.32 [32]. After the quality control (QC) process, paired-end sequence data were combined using PandaSeq [33]. Primers were then trimmed using a proprietary program of ChunLab using a similarity cut-off of 0.8. Sequences were denoised using mothur’s pre-clustering algorithm, which combines sequences and extracts distinct sequences with two or more differences [34]. The EzTaxon database was blasted using BLAST 2.2.22 for taxonomic assignment [35], and pairwise alignment [36] was utilized to detect chimeras for reads that contained a lower than 97% best hit similarity rate [37]. Sequence data were then clustered by CD-Hit [38] and UCLUST [39], and alpha and beta diversity analysis expressed with OTUs [40], Chao1 [41], ACE [42], Shannon [43] were carried out using BIOiPLUG, which is ChunLab’s bioinformatics cloud platform.

### Statistical methods

All data obtained in this study were analyzed using SPSS version 22.0 for Windows (SPSS Inc., Chicago, USA). Dietary and relative abundance of gut microbiota data were visualized with R statistical package, version 3.4.4. The characteristics, body composition, and gut microbiota data of the participants are presented as means and standard deviations, and dietary intake data are presented as medians and inter-quartile ranges. Kruskal-Wallis tests with Bonferroni post hoc tests were used to determine the significance of the differences in gut microbiota composition, alpha diversity, body composition, and dietary intake among groups. The correlations between characteristics of the gut microbiota, dietary intake, and body composition were analyzed using Spearman’s rank correlation analysis. The significance level (α) of all statistical analyses was set to 0.05.

## Results

### Body composition

There were significant differences in body composition according to exercise type and dietary habits. The body compositions of all subjects are presented in Table 1. Lean tissue was the highest in bodybuilders (70.4 ± 9.2 kg) and lowest in distance runners (51.8 ± 4.1 kg) (bodybuilders versus distance runners: *P* < 0.001). Fat tissue was the highest in controls (19.4 ± 7.9 kg) and lowest in distance runners (5.5 ± 1.1 kg) (controls versus distance runners: *P* < 0.001). Body fat percentage was the highest in controls (23.9 ± 6.9%) and lowest in distance runners (9.2 ± 1.6%) (controls versus distance runners: *P* < 0.001). The body composition characteristics and career of each group confirmed that both bodybuilders and distance runners were adapted to specific exercise stimuli for a long time.

### Dietary intake

**Fig. 1:**
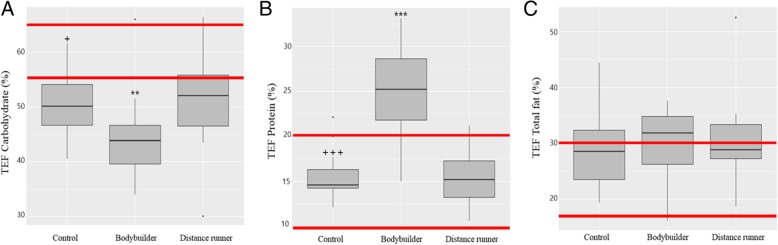
Comparison of the percentage of energy from each macronutrient category. The percentages were calculated by dividing the available energy from the macronutrients by the total energy. **a** The percentage of the energy obtained from carbohydrates; **b** The percentage of the energy obtained from protein; **c** The percentage of the energy obtained from total fat. The red line represents acceptable macronutrient distribution ranges (AMDR). The AMDR for carbohydrates is 55 to 65%, for total fat is 15 to 30%, and for protein is 7 to 20% of the energy intake for Korean adults. Controls versus bodybuilders: ^+^*p* < 0.05 or ^+++^*p* < 0.001, bodybuilders versus distance runners: ^**^p < 0.01 or ^***^p < 0.001

Macronutrient intake of the subjects is shown in Table 2. Athletes consumed more energy than controls; specifically, carbohydrate and lipid intake was significantly higher in athletes than controls. Protein intake was not significantly different between the controls and the distance runners, but bodybuilders consumed significantly more protein than the other two groups. None of the groups ate the recommended daily amount of dietary fiber (> 25 g). Regarding the protein:carbohydrate ratio, the bodybuilders were twice as high as the other groups (*P* < 0.001), and regarding the dietary fiber:carbohydrate ratio, the controls were 1.4-times higher than the distance runners (*P* < 0.05). The nutrient uptake ratios showed that the bodybuilders had a high protein diet pattern and the distance runners had a low dietary fiber diet pattern. The energy ratio of macronutrients of the three groups and dietary reference intakes for Koreans are shown in Fig. 1 (carbohydrate: 55–65%; protein: 7–20%; total fat: 15–30%). The energy contribution ratio of carbohydrates of all groups was lower than that of dietary reference intakes for Koreans, and the bodybuilder group was significantly lower than controls (*P* < 0.05) and distance runners (*P* < 0.01). The energy contribution ratio of protein and total fat of controls and distance runners fulfilled dietary reference intakes for Koreans, and bodybuilders exceeded it. The percent of energy from protein among the bodybuilders was significantly higher than the other groups (*P* < 0.001) but the percent of energy from total fat did not differ between groups.

**Table 2:** Composition of the macronutrients and dietary fiber intake of controls, bodybuilders, and distance runners obtained from 3-d food diaries

	DRIK	Controls (*n* = 15)	Bodybuilders (*n* = 15)	Distance runners (*n* = 15)
Energy (kcal)	2600	1757^+++^/2046–1306	3362 /5392–2488	2787^¥¥¥^/3756–2371
Carbohydrates (g)		219^+++^/ 264–160	411 / 547–319	369^¥¥¥^/ 408–325
Protein (g)		71^+++^/ 84–51	236***/ 314–175	103/ 111–79
Total fat (g)		49^+++^/ 85–39	131 / 188–76	88^¥¥^/ 120–65
Saturated fat (g)	< 20 g	10^+++^/ 11–8	4** / 7–3	14 / 18–9
Monounsaturated fat (g)		11^++^/ 13–10	5** /12–5	16 / 18–9
Polyunsaturated fat (g)		9 /11–7	5 /12–5	9 / 14–5
Dietary fiber (g)	≥25 g	14^+^/ 19–11	19 / 36–14	17 / 20–15
Protein: Carbohydrate ratio		0.3^+++^	0.6^+++^	0.3
Dietary fiber: Carbohydrate ratio		0.07	0.06	0.05^¥^

Data shown as median/IQR (Q3-Q1), controls versus bodybuilders: ^+^*p* < 0.05, ^++^*p* < 0.01 or ^+++^*p* < 0.001, bodybuilders versus distance runners: **p < 0.01, ***p < 0.001, distance runners versus controls: ^¥¥^*p* < 0.01, ^¥¥¥^*p* < 0.001. DRIK: Dietary Reference Intakes for Koreans

### Microbial taxonomy

**Fig. 2:**
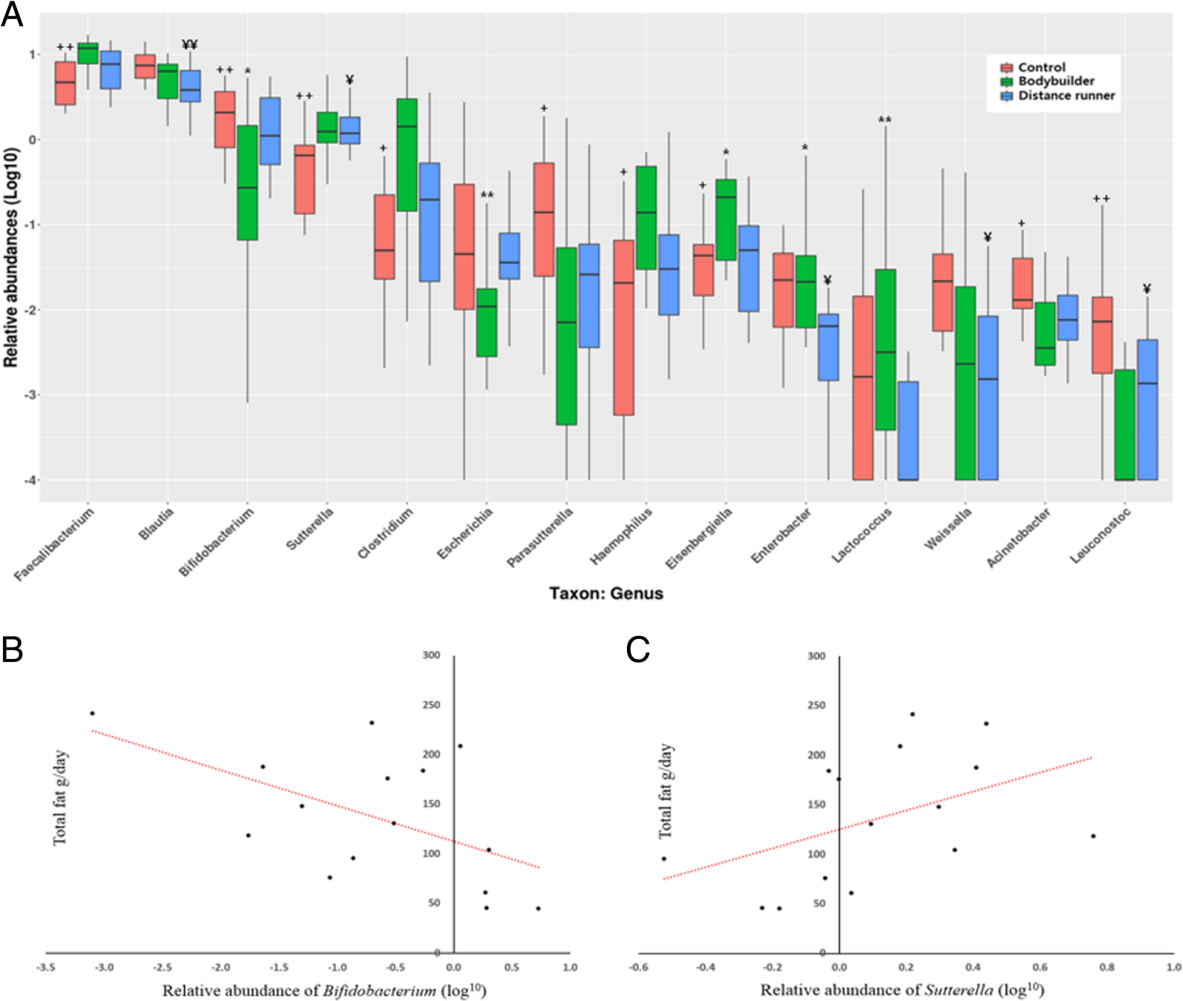
Certain types of exercise training and athlete diet affected the relative abundance of some microorganisms. **a** Comparison of gut microbiota relative abundance at the genus level in the three groups. Relative abundance represent log of percentage as whole microbiota. For example, when the relative abundance of a particular genus is 1, it means 10% of whole microbiota, and when it is 0, it means 1% and when it is −1, it means 0.1%. Controls versus bodybuilders: ^+^*p* < 0.05 or ^++^*p* < 0.01, bodybuilders versus distance runners: ^*^p < 0.05 or ^**^p < 0.01, distance runners versus controls: ^¥^*p* < 0.05 or ^¥¥^*p* < 0.01. **b** Total fat intake negatively correlated with relative abundance of *Bifidobacterium* in bodybuilders (Correlation coefficient: −0.52, p-value: 0.048). **c** Total fat intake positively correlated with relative abundance of *Sutterella* in bodybuilders (Correlation coefficient: 0.58, *p*-value: 0.023)

Type of exercise training and athlete diet influenced relative abundance of gut microbiota at the genus and species levels. *Faecalibacterium*, *Sutterella*, *Clostridium*, *Haemophilus,* and *Eisenbergiella* were the highest (*P* < 0.05) in bodybuilders while *Bifidobacterium* and *Parasutterella* were the lowest (*P* < 0.05) in bodybuilders (Fig. 2a). In particular, the high fat intake by bodybuilders was related to the relative abundance of *Bifidobacterium* and *Sutterella*. The relative abundance of *Bifidobacterium* in bodybuilders was negatively correlated with fat intake (*r* = − 0.52, *p* = 0.048) (Fig. 2b), while the relative abundance of *Sutterella* in bodybuilders was positively correlated with fat intake (*r* = 0.58, *p* = 0.023) (Fig. 2c). At the species level, intestinal beneficial bacteria, which are widely used as probiotics (*Bifidobacterium adolescentis* group, *Bifidobacterium longum* group*, Lactobacillus sakei* group), and those producing short chain fatty acids (*Blautia wexlerae, Eubacterium hallii*) were the lowest (*P* < 0.05) in bodybuilders and the highest (*P* < 0.05) in controls (Table 3).

**Table 3:** Gut microbiota composition at species level

	Controls (*n* = 15)	Bodybuilders (*n* = 15)	Distance runners (*n* = 15)
*Blautia wexlerae*	4.9 ± 2.6^**+**^	3.2 ± 1.9*	2.1 ± 1.1^¥¥^
*Faecalibacterium prausnitzii*	2.3 ± 2.0	4.9 ± 3.8	2.8 ± 2.4
*Eubacterium hallii*	2.0 ± 1.1^**+**^	1.0 ± 0.7	1.2 ± 0.9^¥^
*Bacteroides stercoris*	1.9 ± 2.6^**++**^	0.4 ± 0.9	0.8 ± 1.8
*B. adolescentis* group	1.3 ± 1.6	0.4 ± 1.1**	1.1 ± 0.9
*Bacteroides caccae*	0.15 ± 0.2	0.2 ± 0.2**	0.9 ± 1.0^¥¥^
*Ruminococcus callidus*	0.3 ± 0.4^**++**^	0.07 ± 0.2*	0.4 ± 0.7
*B. longum group*	0.5 ± 0.5^**++**^	0.1 ± 0.1	0.3 ± 0.3
*Escherichia coli* group	0.4 ± 0.8^**+**^	0.2 ± 0.8**	0.3 ± 0.7
*Faecalibacterium_uc*	0.1 ± 0.06^**+++**^	0.2 ± 0.1	0.2 ± 0.1
*Sutterella_uc*	0.03 ± 0.03^**++**^	0.07 ± 0.05	0.07 ± 0.06^¥^
*Bifidobacterium_uc*	0.08 ± 0.03^**+**^	0.03 ± 0.04*	0.07 ± 0.06
*Clostridium innocuum* group	0.1 ± 0.3	0.007 ± 0.02	0.002 ± 0.004^¥^
*Weissella confusa* group	0.07 ± 0.1	0.03 ± 0.1	0.001 ± 0.002^¥¥^
*Enterobacter cloacae* group	0.03 ± 0.02	0.08 ± 0.2**	0.005 ± 0.005^¥¥^
*Lactobacillus sakei* group	0.05 ± 0.06^**+**^	0.01 ± 0.03	0.03 ± 0.05

Data shown as mean ± SD, controls versus bodybuilders: ^+^*p* < 0.05 or ^++^*p* < 0.01 or ^+++^*p* < 0.001, bodybuilders versus distance runners: *p < 0.05 or **p < 0.01, distance runners versus controls: ^¥^*p* < 0.05 or ^¥¥^*p* < 0.01 or ^¥¥¥^*p* < 0.001

### Microbial diversity

**Fig. 3:**
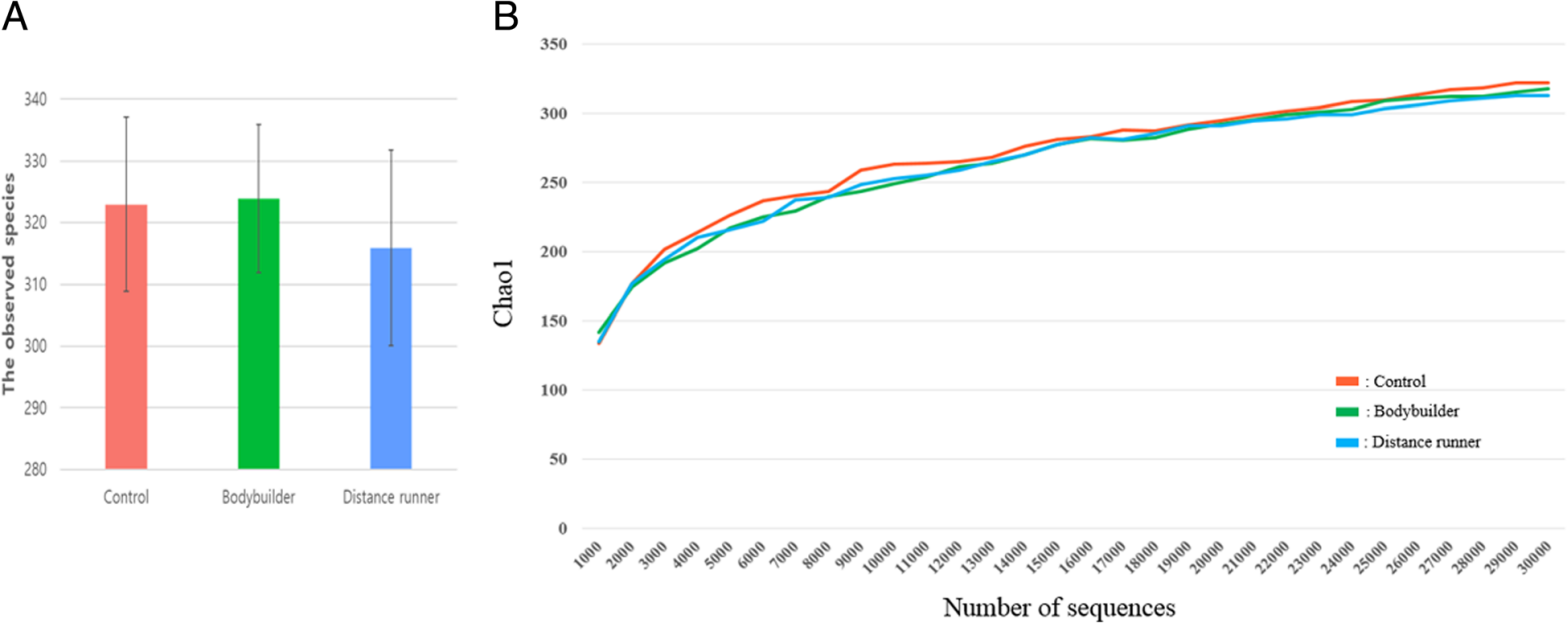
There was no difference in the gut microbiota diversity between the controls, bodybuilders, and distance runners. **a** Comparison of observed species of controls, bodybuilders, and distance runners obtained from 30,000 sequences per sample. **b** Estimation of the abundance of unique operational taxonomic units (OTUs) using Chao1. Phylogenetic diversity was estimated using the average values for the Chao1 plot of the gut microbiota in the controls, bodybuilders, and distance runners. Data are based on 30,000 sequences per sample from the study subjects

**Fig. 4:**
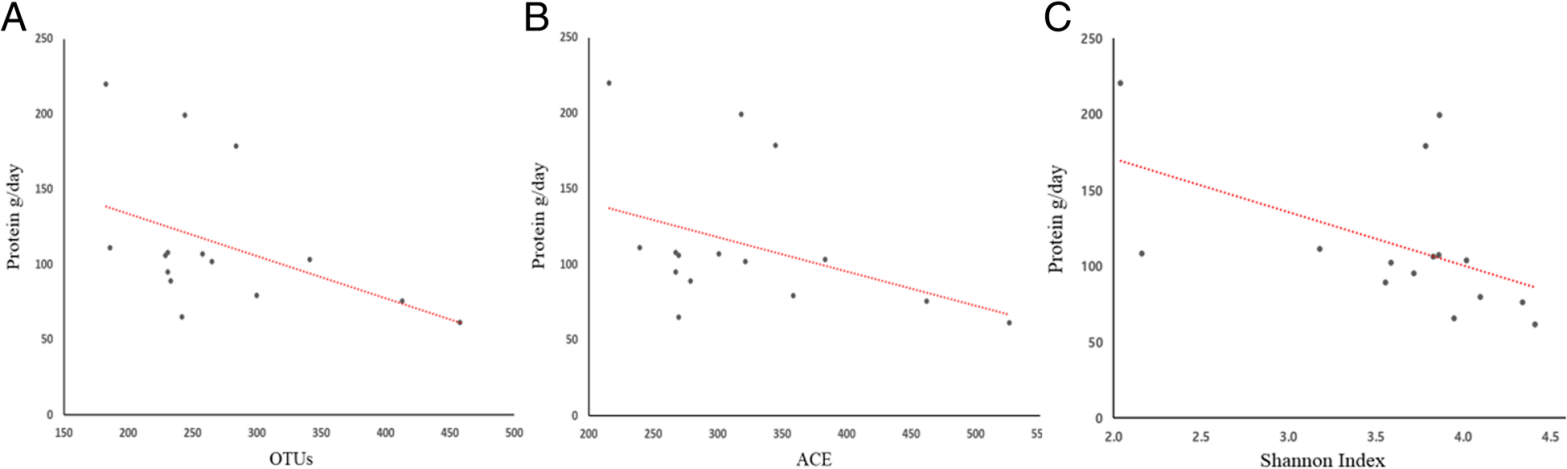
Protein intake negatively correlated with alpha diversity in distance runners. **a** Protein intake negatively correlated with OTUs (Correlation coefficient: − 0.53, *p* value: 0.04). **b** Protein intake negatively correlated with ACE (Correlation coefficient: − 0.51, p-value: 0.05). **c** Protein intake negatively correlated with Shannon index (Correlation coefficient: − 0.63, p-value: 0.012)

**Fig. 5:**
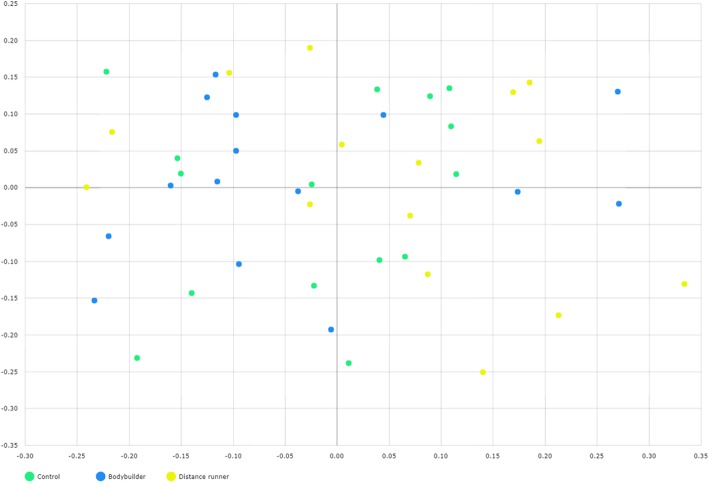
The plot was generated using a generalized UniFrac principal coordinate analysis (PCoA) of fecal microbiota from 45 subjects. Generalized UniFrac PCoA analyzed genus rank level and included unclassified OTUs. Subject color coding: green, controls; blue, bodybuilders; and yellow, distance runners

Aerobic or resistance exercise training accompanied by an unbalanced intake of macronutrients and low intake of dietary fiber did not lead to increased diversity of gut microbiota (Fig. 3a and b). Specifically, for distance runners, a negative correlation was found between protein intake and gut microbiota diversity indices (Fig. 4). Daily protein intake showed a negative correlation with operation taxonomic unit (*r* = − 0.53, *p* = 0.04), ACE (*r* = − 0.51, *p* = 0.05), and Shannon index (*r* = − 0.64, *p* = 0.01) in distance runners. Despite differences in exercise type, body composition, and nutrient intake, the gut microbiota beta diversity of healthy men in the control group and the athlete groups did not differ (Fig. 5).

## Discussion

There are several reports regarding the effects of various nutrients and diet patterns on human gut microbiota [23, 44, 45] but, recently, physical exercise was disclosed as yet another factor affecting the composition, diversity, and metabolic activity of the gut microbiota [15, 17, 18]. However, the impact of physical exercise associated with diet pattern and type of exercise training on the gut microbiota is not fully understood. Our findings, in contrast to recent studies, indicate that type of exercise training and the diet pattern associated with specific sports did not make a difference in the beta diversity of gut microbiota, but they did affect the relative abundance of certain intestinal microbes. In particular, bodybuilders’ high fat intake made *Sutterella* more abundant, while significantly reducing the abundance of *Bifidobacterium*. Cani et al. (2007) reported that a high fat diet caused a decrease in *Bifidobacterium* [46], and *Bifidobacterium* has a negative correlation with the concentration of lipopolysaccharide (LPS), an endotoxin, in the blood [46]. A high protein, low carbohydrate (HP-LC) diet in conjunction with a high fat diet causes a decrease in *Bifidobacterium* [47, 48]. The lack of *Bifidobacterium* resulting from an HP-LC diet could be due to a shortage of carbohydrate-based substrates, the detrimental effects of protein-fermentative metabolites, or competitive exclusion by protein-fermenting microbes in the gut [49]. Several reports have accentuated the significance of *Bifidobacterium* in modulating intestinal homeostasis, regulating local and systemic immune responses, and defending against inflammatory diseases and infections [50, 51]. In addition, acetate-producing bacteria (e.g., *Blautia wexlerae*, *Bifidobacterium adolescentis* group, and *Bifidobacterium longum group*) and lactate-producing bacteria (e.g., *Lactobacillus sakei* group) appeared less in bodybuilders, which may have led to the observed decrease in butyrate-producing *Eubacterium hallii*, which use acetate and lactate as substrates. *Eubacterium hallii* is one of the most abundant butyrate-producing bacteria in the human intestine (followed by *Faecalibacterium prausnitzii*, *E. rectale*, *E. hallii*, and *Anaerostipes hadrus*) [52, 53]; it uses glycerol to produce reuterin, an antimicrobial substance that regulates the homeostasis of intestinal microbial metabolism and inhibits pathogens [54]. As a result, the HP-LC and high fat diet of the bodybuilders is expected to lower the relative abundance of *Bifidobacterium* and, in particular, to lower the relative abundance of acetate-producing bacteria and lactate-producing bacteria, thus influencing the substrate supply of butyrate-producing bacteria. *Sutterella*, highly expressed in athletes, is a genus of Gram-negative, anaerobic, nonspore-forming bacteria that is associated with autism, Down’s syndrome, and inflammatory disease [55, 56]. *Sutterella* is augmented by a high-fat diet as well as by a low-fiber diet [57]. The mechanism of the increase of *Sutterella* may due to the decrease in mucosal thickness caused by the lack of luminal butyrate, because it can adhere to epithelial cells [58]. Therefore, the increase of *Sutterella* in athletes may be associated with the lack of butyrate-producing bacteria such as *Bifidobacterium adolescentis* group, *Bifidobacterium longum* group, *Blautia wexlerae*, *Lactobacillus sakei* group, and *Eubacterium hallii*.

Athletes who had adapted to certain exercise training practices and diet regimens for long time periods did not have the high gut microbiota diversity found in controls who did not engage in regular exercise training. Specifically, in the case of low carbohydrate and dietary fiber intake of distance runners, gut microbiota diversity tended to decrease as protein intake increased. However, Clarke et al. (2014) reported that as protein intake in rugby athletes increased, the gut microbiota diversity also increased. This inconsistency between our results and those of Clarke et al. seems to be caused by the differences in nutrition status of the athletes [18]. The rugby athletes of Clarke et al.’s study met all of the recommended intake requirements, while the athletes of our study had insufficient carbohydrate and dietary fiber intake. Carbohydrates and dietary fiber are the main nutrients that provide carbon and energy to the intestinal microorganisms. In particular, adequate intake of dietary fiber increases the diversity of gut microbiota [59, 60]. Therefore, inadequate intake of carbohydrates and dietary fiber by athletes seem to counteract the benefits of exercise and a high protein diet that tend to increase gut microbiota diversity. Taken together, our results suggest that high-protein diets may have a negative impact on gut microbiota diversity for athletes in endurance sports who consume low carbohydrates and low dietary fiber, while athletes in resistance sports that carry out the HP-LC and high fat diet demonstrate a decrease in short chain fatty acid-producing commensal bacteria. Additional studies should be conducted to determine the effects of external stimuli on the gut microbiota characteristics, exercise performance, and physical condition in athletes.

## Abbreviations

BMI: Body Mass Index
DRIK: Dietary Reference Intakes for Koreans
DXA: Dual-energy X-ray Absorptiometry
HP-LC: High Protein, Low Carbohydrate
IPAQ: International Physical Activity Questionnaire
LPS: lipopolysaccharide
MET: Metabolic Equivalent of Task
